# Scurvy masquerading as IgA vasculitis

**DOI:** 10.1186/s12969-024-00992-2

**Published:** 2024-05-18

**Authors:** Hanna L. Kassa, S. Singh, M. Douglas-Jones, Gill Schermbrucker, J De Lange, Frank Phoya, Claire Butters, Carol Hlela, Ashton Coetzee, Ebrahim Banderker, Kate Webb

**Affiliations:** 1 Department of Pediatric Rheumatology, Red Cross Children’s Hospital, University of Cape Town, Cape Town, South Africa; 2 National Health Laboratory Service, Red Cross Children’s Hospital, University of Cape Town, Cape Town, South Africa; 3 Department of Maxillofacial and Oral Surgery, Groote Schuur and Red Cross Children’s Hospital, University of the Western Cape, Cape Town, South Africa; 4 Victoria Hospital, Wynberg, Cape Town, South Africa; 5 Red Cross Children’s Hospital, University of Cape Town, Cape Town, South Africa; 6 Department of Paediatric Nephrology, Red Cross Childrens Hospital, University of Cape Town, Cape Town, South Africa; 7 Red Cross Children’s Hospital, University of Cape Town, Cape Town, South Africa

**Keywords:** Scurvy, Vitamin-C, Purpuric-rash, Selective-diet, IgA vasculitis, Henoch Schonlein Purpura

## Abstract

**Background:**

Vitamin C deficiency, or scurvy, is rare but poses risks for children with poor diets, limited resources, or malabsorption issues. It may also be common in children with restrictive or selective dietary habits in children with global developmental delay, autism spectrum disorder, and physical disabilities. Symptoms include fatigue, irritability, joint and muscle pain, joint swellings, edema, swollen gums, easy bruising, and delayed wound healing. Early recognition and prompt intervention are essential to prevent the progression of symptomatic vitamin C deficiency in children.

**Case presentation:**

We present a case of a 13-year-old boy with developmental delay secondary to Lennox Gastaut syndrome referred for suspected recurrent, severe, and atypical IgA vasculitis. He presented with irritability, loss of appetite, petechial and ecchymotic lower limb lesions, unilateral gum swelling, severe arthritis, peripheral oedema, severe weight loss, anaemia, and raised inflammatory markers. Multiple investigations were performed before the diagnosis of scurvy was made. A surgical finding of friable gingival tissue with multiple loose teeth, a skin biopsy with follicular hyperkeratosis and extravasated perifollicular red blood cells, and a typical X-ray finding led to the diagnosis of scurvy.

**Conclusion:**

Scurvy should be given careful consideration as a differential diagnosis in patients presenting with musculoskeletal issues, mucocutaneous complaints, and constitutional symptoms such as malaise, asthenia, irritability, and loss of appetite. A focused and detailed dietary history looking for a lack of good sources of vitamin C can be an easy indicator of this differential.

Imaging studies revealing the typical features can also help make the diagnosis. Pathology of the skin revealing pathognomonic features can add to the certainty of the diagnosis. In the absence of all else, the rapid response to treatment with an appropriate dose of vitamin C has a diagnostic and therapeutic role.

## Background

Scurvy, first mentioned by the ancient Egyptians in the ancient Papyrus of Ebers in 1550 BC, is regarded as an ancient disease. It affected sailors from the 15th to the 18th centuries during the Ages of Exploration, due to the limited availability of fresh fruits and vegetables during protracted sea trips. Following the identification of vitamin C's role in scurvy prevention, the illness became uncommon globally. However, cases are reported in modern times in children with autism, developmental and neurological disorders, psychiatric disorders, and extremely fussy eating, and therefore the diagnosis should be considered in this setting [1–4].

Vitamin C is a necessary molecule for the formation of collagen, which is a structural component of blood vessels, skin, bone, cartilage, and teeth. In its reduced form, ascorbic acid is a potent antioxidant that shields vital biomolecules like proteins, lipids, carbohydrates, and nucleic acids from oxidative harm from exposure to toxins and pollutants. It also contributes to the immune system and iron absorption [5, 6].

A lack of vitamin C disrupts collagen synthesis, causing deformity and a lack of mature collagen. This causes capillary brittleness, which can result in cutaneous hemorrhage, mucosal swelling, hemarthroses, limb and joint swelling, myalgia, and arthralgia. Vitamin C deficiency causes osteoid matrix production errors. This causes osteopenia, bone fragility, epiphyseal fractures, subperiosteal hemorrhage, bone pain, and difficulty walking. Additionally, it results in anemia, which can eventually contribute to pulmonary hypertension. Prostaglandin metabolism can also be dysregulated due to a vitamin C shortage, which can cause inflammation and increased inflammatory markers. This results in anorexia, irritability, and failure to thrive [7, 8].

Scurvy symptoms often appear one to three months after poor dietary vitamin C consumption [9, 10]. The first symptoms to appear were historically illustrated as irritability and easy fatigability with a craving for rest. This is followed by bleeding gums and a red rash on the skin, with associated poor healing of minor wounds on the body [11]. Later, overt signs of inflammatory arthritis, pedal edema, bone pain with failure to walk, and a worsening appetite ensue. We present here a case of scurvy initially misdiagnosed as an IgA vasculitis due to mimicking clinical signs.

## Case presentation

A 13-year-old male with global developmental delay and epilepsy secondary to Lennox Gastaut syndrome presented to his local general pediatrics service in March 2022 with a bilateral lower limb purpuric rash, arthritis, severe anemia (Hb=5.9g/dl), normal platelets (368x10^9^/L), elevated C reactive protein (CRP=80mg/L) and elevated serum IgA (5.6 g/L range 0.5-3.5g/L) (Table 1). In the peripheral hospital, due to the presence of lower limb purpura and arthritis, the child was diagnosed with IgA vasculitis and managed conservatively [12].

**Table 1:** Clinical Laboratory tests

**Test**	**Normal range**	**March 2022**	**February 2023**	**Admission March 2023**	**April 2023**
WBC	3-10 x10^9^/L	7.1	6.4	4.1	4.4
Hg	13-17g/dl	5.9^a^	9.5^a^	4^a^	9.3^a^
MCV	83-101fL		81	75	95
RDW	12-16%		14.5	17.6	21
PLT	171-388 x 10^9^/L	368	352	278	395
CRP	<10mg/L	80^a^	47^a^	89^a^	15^a^
ESR	3-13mm/hr	42﻿^a^	79﻿^a^	42﻿^a^	
Cr	40-72 umol/L	40	44	51	
Urea	2-6mmol/L	3.3	2.5	5.1	
Urine P:Cr	<0.015 g/mmol		0.23^a^	0.041^a^	
Albumin	32-47g/L	31﻿^a^	33	29﻿^a^	34

^a^Represents abnormal result

After an initial improvement, he presented again a year later in February 2023, with a lower limb purpuric rash and arthritis. He had moderate anemia (Hb=9.5 g/dl), raised acute phase reactants (CRP-47mg/L, ESR-79mm/hr) normal platelets (352 x10^9^/L), normal INR (1.18) and a raised urine protein to creatinine ratio (Pr:Cr=0.23 g/mmol). Again, he was diagnosed with recurrent IgA vasculitis. Three weeks later, an oral mass was noted. He was given amoxicillin-clavulanic acid and referred to the pediatric nephrology and rheumatology teams at the Red Cross Children’s Hospital for further workup of incalcitrant IgA vasculitis.

Upon admission, he was noted to have an additional 3-week history of progressive lower limb bruising, pain and swelling, poor feeding, and irritability, and he had recently stopped walking. His caregivers complained of the mass in his lower jaw that was growing and becoming disfiguring to his face.

Past medical history revealed that he was a twin, born at term, and that he had developed multiple seizures as an infant. His parents were known to abuse alcohol and amphetamines, and they had nine older children. Due to these circumstances, he and his twin were placed in the care of their elderly grandparents from birth. His seizures were well controlled with phenobarbitone (6 mg/kg/day), valproic acid (40 mg/kg/day), risperidone (0.06 mg/kg/day), and lamotrigine (5 mg/kg/day). He was unable to communicate verbally and was unable to perform self-care activities like feeding, dressing, and toileting independently. His twin brother had normal neurological development. The family received a care dependency grant from the government, and he attended a special-needs school. According to his grandfather, he normally ate cooked porridge, noodles, cabbage, and bananas and required a long time and a lot of support to feed.

**Fig. 1:**
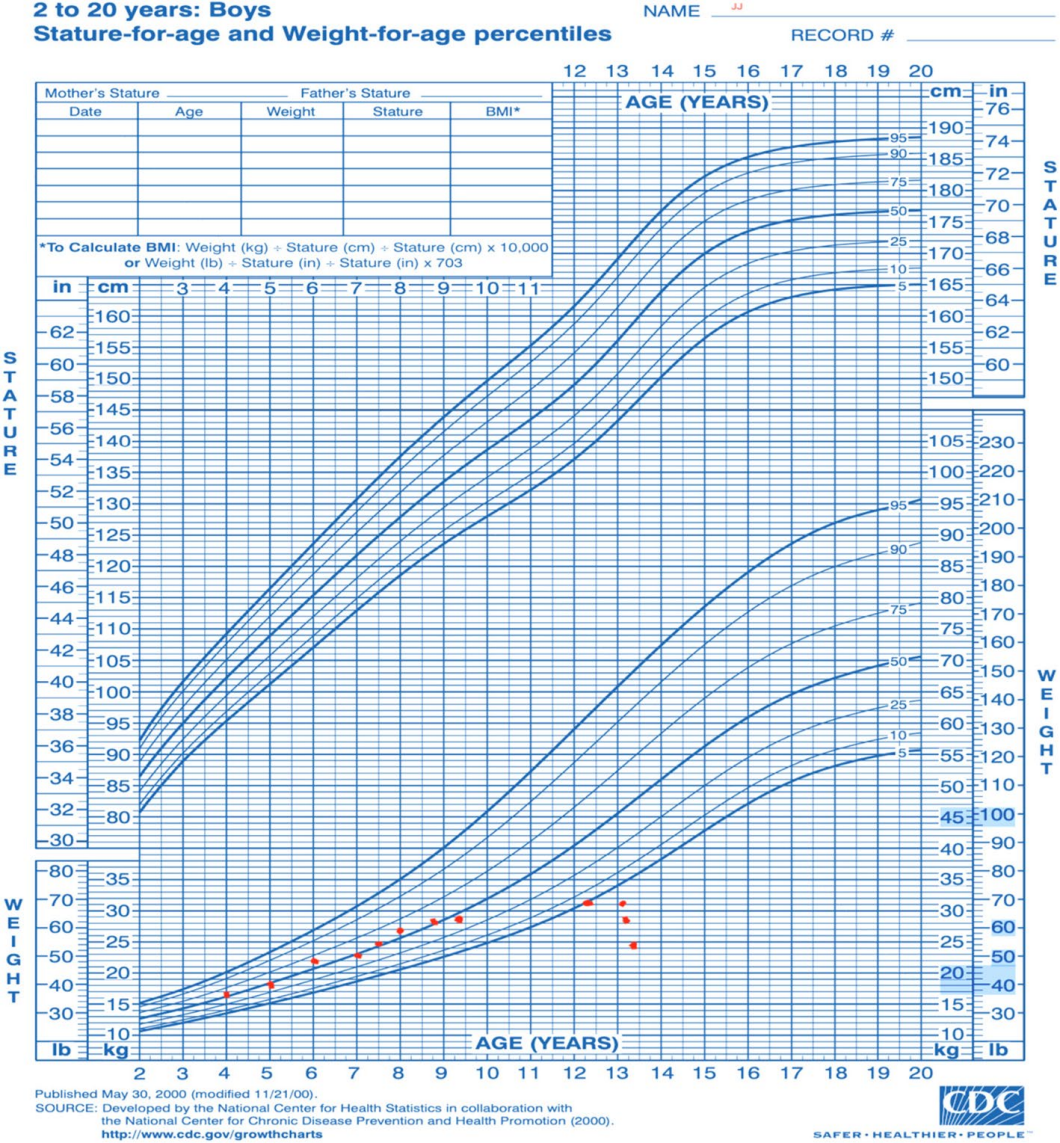
Weight for age percentile CDC 2-20 years chart for boys [26]: child’s plot shows a stagnant weight from age 9 to 13, making him underweight, with a later rapid dip in his weight over 4 months

**Fig. 2:**
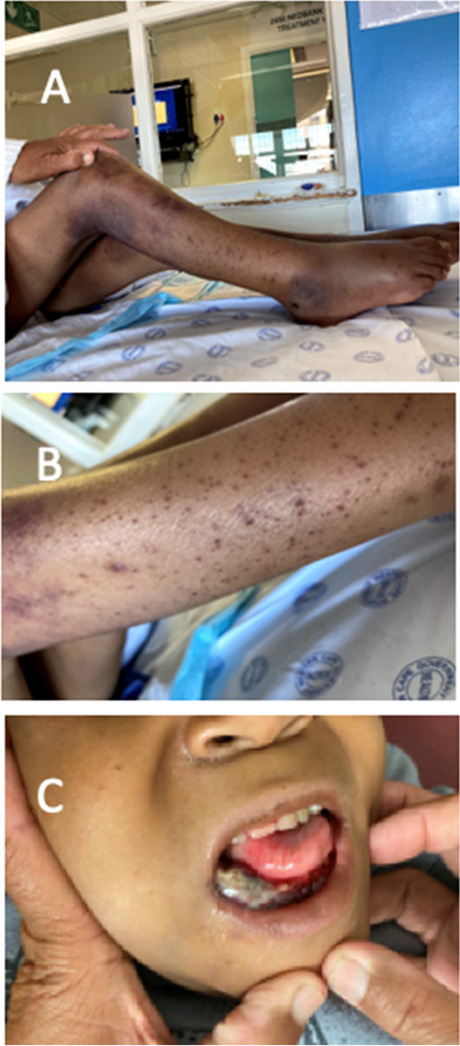
Child’s mucocutaneous condition on admission: shows areas of ecchymosis, diffuse petechial rash, and pedal edema with right-sided intraoral mass disfiguring the face

His physical exam revealed a severely underweight child (24.5kg below the 5^th^ centile for age) with rapidly progressive weight loss for 4 months (Fig. 1). He could not communicate and was irritable, in pain, and lying flexed in his bed. His blood pressure was 103/70mm Hg, and his heart rate was 110 beats per minute. He had an open mouth with a large right-sided intraoral mandibular fungating and protruding mass with no active bleeding or discharge. He also had petechial and ecchymotic lesions on his upper and lower limbs (Fig. 2). There was bilateral pitting pedal and pretibial edema with effusions, pain, and limitation of his bilateral ankles, knees, and right wrist.

He had severe anemia (Hb = 4 g/dl), leukopenia (1.8 x10^9^/L), elevated CRP (89 mg/dl), elevated erythrocyte sedimentation rate (ESR = 42 mm/hr), and a normalizing urine protein to creatinine ratio (0.041 g/mmol). Anti-nuclear antibody, anti-proteinase 3 antibody, and anti-myeloperoxidase antibody were negative. Complement 3 and 4 levels done previously when the child was having proteinuria were normal (C3-1.5, 0.9 to 1.8; C4-0.28, 0.1 to 0.4) and not repeated since proteinuria was improving.

**Fig. 3:**
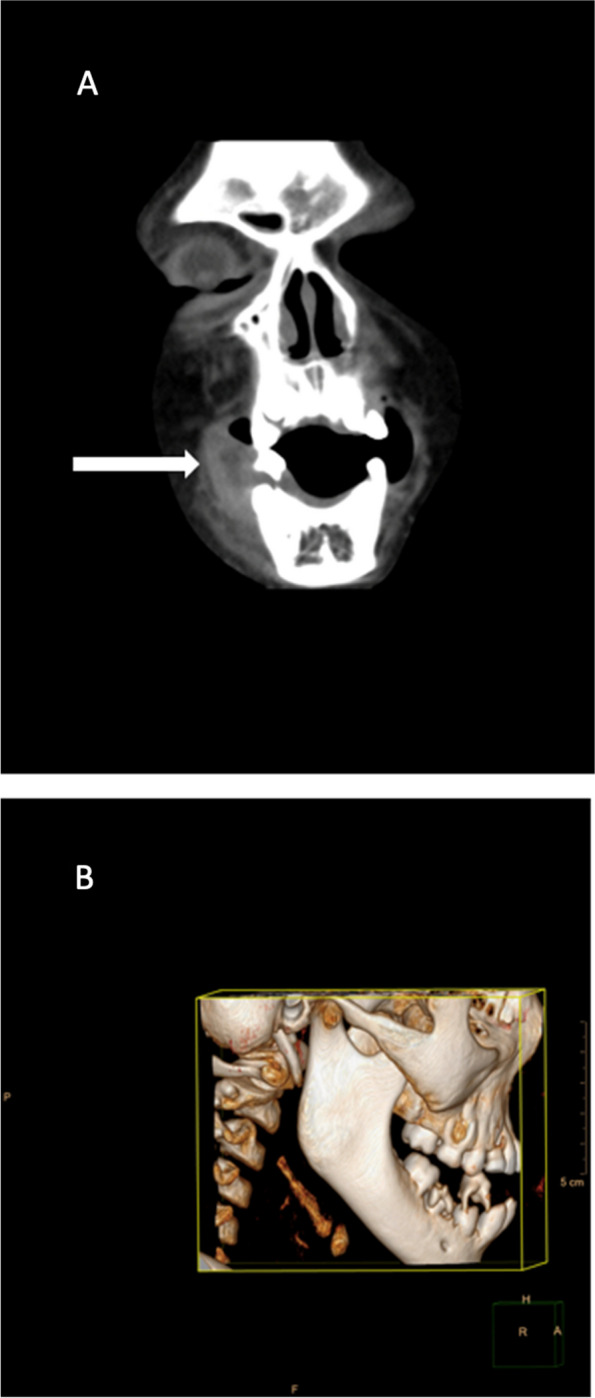
Un-contrasted CT scan of patient’s mandible: shows right peri-mandibular soft tissue swelling with a central low density suggestive of an inflammatory mass with central necrosis and no signs of destructive bone changes

A computed tomography (CT) scan of the jaw mass revealed an inflammatory mass or abscess evident both externally as a facial swelling and intraorally as a mass with no bony destruction (Fig. 3). Maxillofacial and Oral Surgery was consulted, and an examination, dental extractions, and biopsy were performed during which gingival friability with hypertrophy and bleeding with deep periodontal pocketing were noted throughout the dentition. All maxillary and mandibular teeth were also noted to be mobile. The retained deciduous teeth were removed, and the gingiva was debrided. Biopsies were taken from the reported intra-oral lesion and two predetermined areas of skin on the upper and lower limbs. After surgery, the surgeons commented specifically on the multiple loose teeth, remarking on the possibility of vitamin deficiency. Histology of the oral mass showed extensive hemorrhage with fibrin and organizing hematoma within the subepithelial stroma. There was focal ulceration and reactive changes within the overlying epithelium. There was no evidence of vasculitis or a vascular neoplasm.

**Fig. 4:**
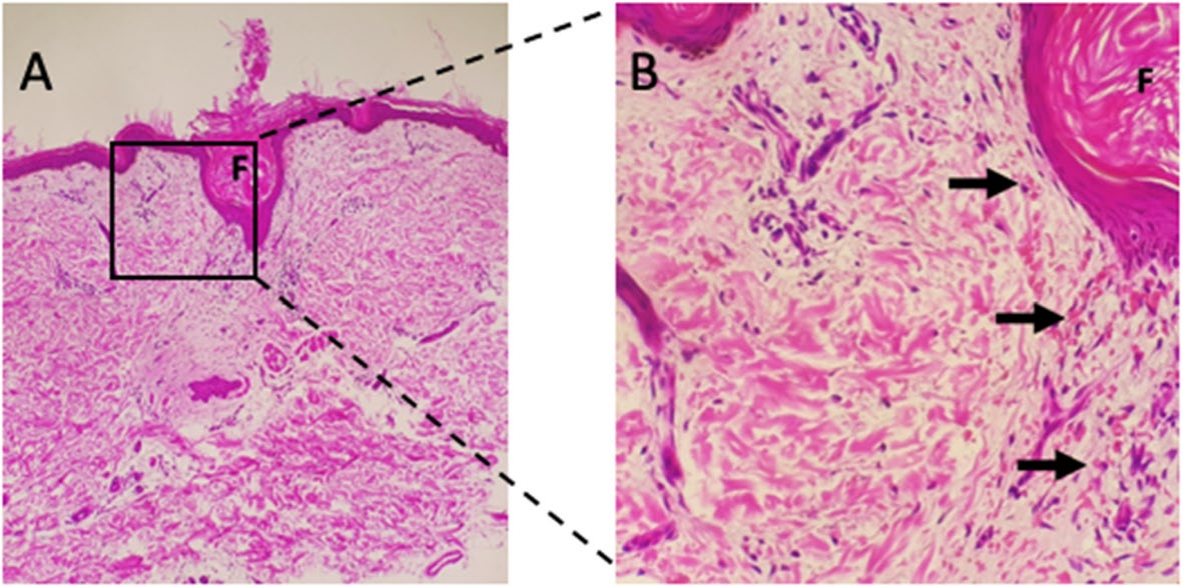
Skin biopsy: Shows epidermal atrophy and follicular hyperkeratosis on low power and perifollicular extravasated red blood cells on high power

**Fig. 5:**
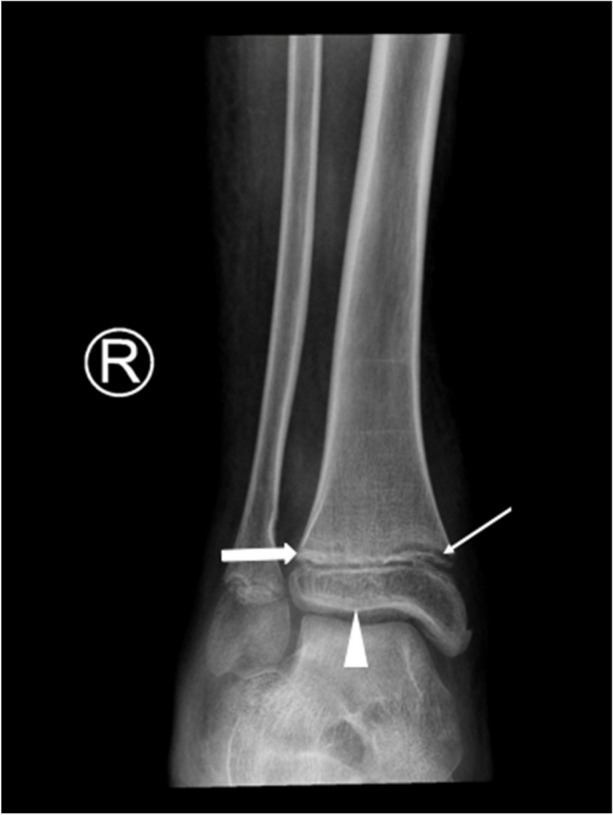
X-ray: X-ray of the right ankle shows Trummerfeld zone, white line of Frankel and Wimberger’s sign

A skin biopsy revealed follicular hyperkeratosis and perifollicular extravasated red blood cells with no associated vasculitis (Fig. 4), which is pathognomonic for vitamin C deficiency. Direct immunofluorescence studies were negative for IgA deposition. Serum level of vitamin C was not available. The serum iron was low, but vitamin D, vitamin B12 and folate were normal. An X-ray of the ankles showed white lines of Frankel, a Trummerfeld zone, and Wimberger’s ring epiphysis (Fig. 5).

In summary, this young boy with Lennox Gastaut syndrome and difficult social circumstances presented with lower limb purpura and arthritis and was initially mis-diagnosed with IgA vasculitis. Upon further weight loss, persistently raised inflammatory markers, anemia, and the development of an intra-oral mass with multiple loose teeth, scurvy was diagnosed clinically.

**Fig. 6:**
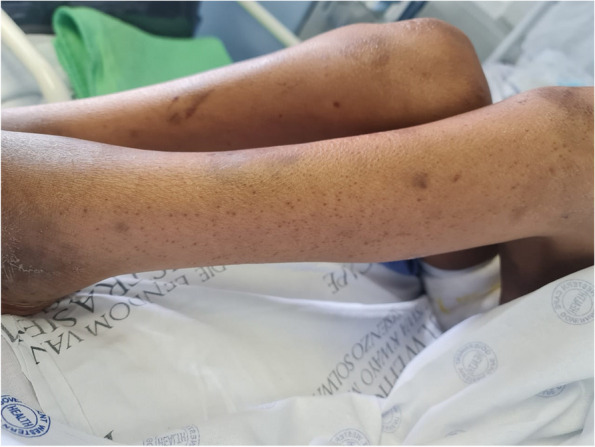
Improved patient’s lower extremities on discharge: shows significant clearing of echymotic and petechial rash

He was treated with vitamin C 100 mg orally three times a day and improved rapidly. His family was provided with dietary advice to enrich his meals with vitamin C, and he was referred to social services for an assessment of his home circumstances. After a week, he had significant improvement in his irritability, appetite, purpuric rash and edema and was discharged (Fig. 6). At two-month follow-up he had gained 4 kg, was walking and mobilizing well and his family reported improved appetite and vigor beyond the previous baseline.

WBC- white blood cells; Hg- hemoglobin; MCV- Mean corpuscular volume; RDW- red cell distribution width; PLT- platelets; CRP- C-reactive protein; ESR-Erythrocyte sedimentation rate; Cr: creatinine; P:Cr- urine protein to creatinine ratio.

## Discussion and conclusions

Here, we describe a case of scurvy in a child, initially misdiagnosed as IgA vasculitis. Poor wound healing, arthralgia, arthritis, petechiae, nonspecific myalgia, gingival bleeding, bruising, and hemorrhage are the main signs of vitamin C insufficiency that might mimic vasculitis. We found three similar reports of scurvy initially misdiagnosed as vasculitis [1, 13, 14]. In the only paediatric report, a 14-year-old girl presented with polyarthralgia, abdominal pain, and purpura, similar to our patient and was thought to have IgA vasculitis until a skin biopsy proved otherwise [1]. Our patient had severe generalized pain, and due to his poor communication capacity, ruling out intermittent abdominal pain was not easy. In retrospect, the fact that our patient had severe anemia without overt gastrointestinal bleeding in a suspected IgA vasculitis patient should have alerted us to alternate diagnosis.

The two other reports we found are from adults; a 58-year-old man and a 62-year-old woman [13, 14]. The 58-year-old presented with purpuric and bullous lower extremity lesions and spontaneous subconjunctival hemorrhage, misdiagnosed on two occasions with cutaneous vasculitis and a bullous drug reaction. A diagnosis of scurvy was finally made with a skin biopsy and a serum vitamin C level [14]. The 62-year-old female patient had progressive bilateral lower extremity pain, weakness, bruising, rash, pancytopenia, and positive myeloperoxidase and proteinase-3 antibodies. Small-vessel vasculitis, systemic lupus erythematosus, and malignancy were considered, and excluded. Scurvy was first suspected after she developed massive abdominal wall echymosis and the diagnosis was confirmed with an undetectable serum vitamin C level [13].

The symptoms of scurvy, as stated above, can be vague and easily mirror those of other systemic disorders, including rheumatologic, neurologic, hematologic, and neoplastic problems. Patients with these symptoms should be evaluated for the possibility of scurvy, especially if they have a history of poor nutrition and consume little to no fresh fruits and vegetables. A thorough dietary history might help distinguish scurvy from other disorders. In the patient presented, the dietary history was reported as adequate, with cabbage, fortified porridge, and bananas included, but on further questioning, it was revealed that the time and support needed to feed adequately was substantial. In addition, the presence of a neurologically unaffected twin with no signs of nutritional deficiency argues for this patient’s neurological and developmental symptoms contributing extensively to his poor nutritional status rather than a lack of access to vitamin C-containing foods.

Although rare in modern times, case reports confirm that children with selective diets secondary to developmental disabilities, feeding difficulties, autism spectrum disorder and eating disorders [4] may be at risk of developing scurvy . Children in poor living conditions with limited access to fresh fruits and vegetables and infants fed purely on unfortified cow’s milk are also at risk [4, 10, 17, 18]. It is important to remember that scurvy also occurs in developmentally healthy and chronic illness-free children that gradually develop a self-restraint towards vitamin C-rich foods [19].

In modern days, where we don’t see many cases of scurvy, a high index of suspicion with a detailed dietary history is required to diagnose vitamin C deficiency. Dietary history should inquire about the intake of vitamin C-rich foods like citrus fruits (including oranges, lemons, and grapefruits), strawberries, kiwi, papaya, mango, pineapple, cantaloupe, watermelon, tomatoes, red and green bell peppers, broccoli, Brussels sprouts, cauliflower, and spinach. Additional good sources are fruit juices, fortified cereals, and supplements. Vitamin C is heat-sensitive and easily damaged by boiling in water and therefore the preparation of meals should also be inquired about. The frequency of intake is important as humans don’t store or synthesize vitamin C, and are dependant on daily intake [20, 21].

With the typical dietary deficiency, children presenting with gingival ulcers, the inability to walk, bone and muscle pain in the lower extremities, and edema are strongly indicative of a vitamin C deficiency.

In scurvy, blood counts commonly show anemia and rarely pancytopenia [22] with elevated acute phase reactants. Serum vitamin C (ascorbic acid) levels below 200 mcg/dl are the gold standard for diagnosis, but may be influenced by recent oral intake, making leukocyte vitamin C levels a better indicator of total body stores. Neither of these tests are readily available in most countries [5, 10].

Classical signs of scurvy on radiographic imaging include the Pelkan spur (zone of provisional calcification extending beyond the margins of the metaphysis, resulting in periosteal elevation and marginal spur formation), the Trummerfeld zone (lucent metaphyseal band underlying the Frankel line), the Wimberger sign (circular, opaque radiologic shadow surrounding epiphyseal centers of ossification, which may result from bleeding), the white line of Frankel (dense zone of provisional calcification), and widespread osteopenia with a pencil-thin cortex. Due to localized hemorrhages, bone marrow edema is also a non-specific x-ray observation in scurvy. These radiographic signs are specific and can significantly aid in determining the diagnosis. The X-ray of our patient showed multiple clear metaphyseal bands in the distal femur called the Trummerfield zone and a thickened zone of calcification called the white line of Frenkel and Wimberger’s epiphyseal ring [11, 23].

Cutaneous vasculitis typically manifests as macular rash that may progress to the pathognomonic ‘palpable purpura’ associated with IgA vasculitis, typically due to the extravasation of blood from vessels, worse over dependent areas such as buttocks and lower limbs [15]. Purpuric lesions may also result from mimics of vasculitis including atheroembolic disease, thrombotic disorders such as antiphospholipid antibody syndrome, thromboembolism or neoplasms such as cardiac myxoma and scurvy [16]. Careful examination and skin biopsy are important to differentiate cutaneous vasculitis from other less common causes of purpura. Pathognomonic signs of scurvy on the histology of the skin include perifollicular hyperkeratosis, perifollicular bleeding, and corkscrew hair. Perifollicular hemorrhages occur as a result of the fragility of small capillaries due to collagen deficiency. This indicates the right site for the skin biopsy to be taken [23–25]. Our patient's skin specimen taken at the site of a petechial lesion showed these typical features, helping to confirm the diagnosis of scurvy despite the absence of serum vitamin C level testing.

In the case of the reported patient, the rapid resolution of symptoms with the intake of vitamin C reassured us that the diagnosis was correct. In less resourced settings where most of the laboratory and pathology investigations needed are not available, a trial of vitamin C supplementation can be the way to diagnose patients with the above-mentioned signs and symptoms.

The dose of vitamin C for treatment used in many case reports is 300mg per day, but it ranges between 100 and 500 mg per day depending on the age and condition of the patient [4, 6, 7, 18]. The duration of treatment depends on the patient’s severity and response to treatment, but many suggest continuing until all clinical signs of vitamin C shortage disappear and then continuing with a diet rich in vitamin C. Common across many case reports is that children show a response to therapy in a few days of treatment and may require a month of supplementation for maximal recovery [5, 8, 10, 23, 25].

In conclusion, although rare, it is important to consider scurvy as a differential diagnosis for musculoskeletal and mucocutaneous complaints, especially in children with neuro-developmental co-morbidities. In addition, in the absence of investigative modalities, a trial of vitamin C supplementation can be used as a diagnostic and therapeutic modality.

## Data Availability

Not applicable.

## Abbreviations

BC: Before Christ
Cr: Creatinine
CRP: C-reactive protein
CT: Computed tomography
ESR: Erythrocyte sedimentation rate
Hg: hemoglobin
IgA: Immune globulin A
MCV: Mean corpuscular volume
P:Cr: urine protein to creatinine ratio
PLT: platelets
RDW: Red cell distribution width
WBC: White blood cells
